# Novel Hematopoietic Target Genes in the NRF2-Mediated Transcriptional Pathway

**DOI:** 10.1155/2013/120305

**Published:** 2013-05-25

**Authors:** Michelle R. Campbell, Mehmet Karaca, Kelly N. Adamski, Brian N. Chorley, Xuting Wang, Douglas A. Bell

**Affiliations:** ^1^Environmental Genomics Section, Laboratory of Molecular Genetics, National Institute of Environmental Health Sciences-National Institutes of Health, Research Triangle Park, NC 27709, USA; ^2^US Environmental Protection Agency, Research Triangle Park, NC 27709, USA; ^3^National Institute of Environmental Health Sciences, Mail Drop C3-03, P.O. Box 12233, Research Triangle Park, NC 27709, USA

## Abstract

Nuclear factor- (erythroid-derived 2) like 2 (*NFE2L2, NRF2*) is a key transcriptional activator of the antioxidant response pathway and is closely related to erythroid transcription factor *NFE2*. Under oxidative stress, NRF2 heterodimerizes with small Maf proteins and binds cis-acting enhancer sequences found near oxidative stress response genes. Using the dietary isothiocyanate sulforaphane (SFN) to activate NRF2, chromatin immunoprecipitation sequencing (ChIP-seq) identified several hundred novel NRF2-mediated targets beyond its role in oxidative stress. Activated NRF2 bound the antioxidant response element (ARE) in promoters of several known and novel target genes involved in iron homeostasis and heme metabolism, including known targets *FTL* and *FTH1*, as well as novel binding in the globin locus control region. Five novel NRF2 target genes were chosen for followup: *AMBP, ABCB6, FECH, HRG-1 (SLC48A1)*, and *TBXAS1*. SFN-induced gene expression in erythroid K562 and lymphoid cells were compared for each target gene. NRF2 silencing showed reduced expression in lymphoid, lung, and hepatic cells. Furthermore, stable knockdown of NRF2 negative regulator KEAP1 in K562 cells resulted in increased *NQO1, AMBP*, and *TBXAS1* expression. NFE2 binding sites in K562 cells revealed similar binding profiles as lymphoid NRF2 sites in all potential NRF2 candidates supporting a role for *NRF2* in heme metabolism and erythropoiesis.

## 1. Introduction

NRF2 (encoded by nuclear factor-erythroid p45-related factor 2, *NFE2L2*) is the master regulator of antioxidant and phase II detoxification genes that collectively resist cellular damage due to electrophilic and oxidative stress [1, 2]. Oxidative stress can be caused by an imbalance between the production of reactive oxygen and the system's ability to detoxify reactive intermediates or repair any damage. Under nonstressed conditions, NRF2, a member of the cap“n”collar family of basic leucine zipper transcription factors, is repressed in the cytosol by Kelch-like ECH-associated protein 1 (KEAP1) [3–6]. In the event of oxidative stress or other stimuli, NRF2 dissociates from KEAP1, translocates into the nucleus, forms heterodimers with small Maf and other bZIP proteins, and binds the NRF2 antioxidant response element (ARE; TGActcaGC) in promoter regions of downstream detoxifying enzymes. Known examples of NRF2 gene targets include hemeoxygenase-1 (*HMOX1*), NAD(P)H: quinone oxidoreductase-1 (*NQO1*), glutamate-cysteine ligase (*GCL*), and glutathione S-transferase (*GST*) [7–10]. Several studies have indicated that NRF2 potentially regulates genes in other pathways such as protein transport, phosphorylation, cell cycle, and growth [11–15]. In addition, chromatin immunoprecipitation (ChIP) deep sequencing of DNA (ChIP-seq) performed in mouse embryonic fibroblasts [16] and human lymphoblastoid cells [17] revealed several hundred novel NRF2-mediated genes that are involved in cellular processes other than oxidative stress such as adipogenesis [17]. In our study, NRF2 was activated in lymphoblastoid cell lines with sulforaphane treatment, a dietary isothiocyanate that acts as a phase II enzyme inducer through the *NRF2* pathway [12]. We identified 849 NRF2 occupied peaks, 242 of which were considered high confidence with 96% of those containing one or more AREs [17]. Putative and novel NRF2-regulated genes were characterized by integrating bioinformatics, expression microarray, additional independent ChIP real-time quantitative polymerase chain reaction (qPCR), and NRF2 short interfering RNA (siRNA) silencing in multiple cell lines for selection of potential candidates for followup. Pathway analysis revealed genes involved in apoptosis, immune response, retinoid signaling, heme metabolism, and iron homeostatis. The potential role of *NRF2* in heme metabolism and iron homeostatis was intriguing since NRF2 is structurally related to nuclear factor-erythroid derived 2 (NFE2), a transcription factor that is essential for erythroid differentiation and is specifically expressed in hematopoietic progenitors and lineages [18]. NFE2 dimerization partners overlap with NRF2 such as small Maf proteins, and NFE2 and NRF2 bind the same AP1-like core motif consensus sequence [17, 19]. In addition, we reported previously [17] that 112 of 242 (~46%) high confidence peaks colocated with ENCODE erythroleukemic cell line K562 NFE2 bound regions, [20] further supporting a possible role of *NRF2* in heme metabolism and erythropoiesis.

Mature erythrocytes or red blood cells (RBCs) are packed with hemoglobin (Hb) which transport oxygen from the lungs to peripheral cells and tissues. Made of four subunits as a tetramer, adult Hb consists of two alpha-globin and two beta-globin chains, each chain consisting of an iron-containing heme group that can bind one oxygen molecule [21–23]. During RBC hemolysis in both normal and disease conditions, Hb can escape resulting in free heme and iron [24, 25]. Excess heme and iron have the ability to damage lipids, proteins, and DNA through oxidation and the production of reactive oxygen species (ROS) which can create a positive hemolytic feedback loop [23]. In addition to the well-studied NRF2 target gene *HMOX1,* which plays a large role in the heme degradation pathway, several known NRF2-regulated genes are involved in iron homeostasis, such as ferritin (*FTH1* and *FTL*). In our ChIP-seq study and in the ENCODE ChIP database, the promoter regions of these genes showed high confidence NRF2-bound peaks with NFE2 peak overlap in K562 cells [17, 26, 27]. Moreover, we saw novel NRF2/NFE2 colocated binding site occupation in the globin locus control region near the *β*-locus for epsilon globin (*HBE1*) and gamma globin (*HBG*) indicating that *NRF2* may be involved in erythroid differentiation (Figure 2(b)).

In order to further assess the relationship between *NRF2* and *NFE2* in erythroid cells, we selected five novel NRF2 candidate target genes identified by overlap between NRF2 ChIP-seq in lymphoid cells and NFE2 ChIP-seq in K562 cells. These genes are known to be involved in heme metabolism and iron homeostasis, and we evaluated their protein and expression levels under a variety of conditions. Candidate NRF2 target genes included *α*_1_-microglobulin/bikunin precursor (*AMBP*), ATP-binding cassette (ABC) transporter 6 (*ABCB6*), ferrochelatase (*FECH*), solute carrier family 48, member 1 (*SLC48A1*) more commonly known in the literature as heme responsive gene 1 (*HRG1*), and thromboxane A synthase (*TBXAS1*). Using our SFN-induced NRF2 ChIP-seq results, we compared ENCODE NFE2 binding peaks, chromatin state (ChromHMM), RNA-seq data, and conservation tracks in order to assess each potential NRF2-regulated candidate. In addition, we activated NRF2 by sulforaphane treatment in K562 cells and compared candidate gene expression in cells of lymphoid or erythroid origin treated with SFN or with NRF2/KEAP1 gene silencing. Candidate genes were also assessed by protein analysis in K562 SFN treated cells as well as in two different stable KEAP1-silenced cell lines. 

## 2. Materials and Methods

### 2.1. Cell Culture

Human lymphoblastoid cells (LCLs; Coriell) were grown in RPMI with 15% fetal bovine serum (FBS; Gemini) and 1X antibiotics/antimycotics (Life Technologies) in 5% CO_2_ at 37°C. Following ATCC recommendations, airway epithelial BEAS-2B (ATCC CRL-9609) and erythroleukemic K562 (ATCC CCL-243) cells were cultured in 10% FBS with 1X antibiotics/antimycotics. HepG2 cells (ATCC HB-8065) were grown in MEM plus 10% FBS, 2.5 mM L-glutamine (Life Technologies), and 1X antibiotics/antimycotics, and A549 cells (ATCC CCL-185) were cultured in Ham's F12K supplemented with 10% FBS and 1X antibiotics/antimycotics. Cells were treated with 0.1% DMSO or 10 *μ*M sulforaphane (SFN) for either 8 (NRF2 siRNA experiments) or 24 hours at 37°C before isolating RNA.

### 2.2. Chromatin Immunoprecipitation (ChIP) and ChIP-Seq

ChIP, sequencing results, and data analysis were previously described [17].

### 2.3. Real-Time Quantitative Polymerase Chain Reaction (qPCR)

RNA was isolated from cells using Qiagen's RNeasy kit per manufacturer's instructions including the on-column DNA digestion step. Quantification of RNA via Qubit (Invitrogen) was followed by cDNA synthesis using the SuperScript First-Strand Synthesis System (Invitrogen). Target amplification was performed in at least triplicate using Applied Biosystems (ABI) primer/probe mixes or custom designed SYBR primers and Universal PCR Master Mix (ABI). Initial fluorescence (Ro value) was calculated using a method of Peirson et al. [28]. Several primers have been previously described [17]. In addition, TaqMan gene expression assays were used for *HRG1* (*SLC48A1*; Hs00215236), *BACT* (Hs99999903), and *18S* (Hs99999901). Custom exon junction spanning SYBR primers were designed for**β*-globin* (Fwd5′-GGTGGTCTACCCTTGGACCC-3′, Rev5′-GATACTTGTGGGCCAGGGCA-3′), *NFE2* specific primers (Fwd5′-CAGAGCAGGAACAGGGTGAT-3′, Rev5′-TGGAGGTCCAAGGTATGGAG-3′), and *TBXAS1* (Fwd5′-CTCCTCTACTGGGTGCAAGC-3′, Rev5′-ATAGCCAGCGATGAGGAAGA-3′). All measurements were reported as target values normalized to *BACT* or *18S* as average values ± standard error of the mean.

### 2.4. NRF2 Gene Silencing (NRF2KD)

NRF2 was silenced transiently in BEAS-2B, HepG2, and A549 cells with a reverse transfection protocol using Ambion siRNAs. Nonspecific scramble control (control; AM4643) and three NRF2 siRNA (ID no. 115764) were tested for NRF2 knockdown by transient transfection using Xtreme Gene (Roche) for BEAS-2B or Lipofectamine 2000 (Invitrogen) reagent for HepG2 and A549 cells following manufacturer's instructions as previously described [17]; ID no. 115764 silencing was most effective and used for further analysis. 

### 2.5. shRNA KEAP1 Knockdown (KEAP1KD)

Mission shRNA lentiviral particles (Sigma) were used to stably silence KEAP1 (SHVRS-NM_012289) in K562 cells following the manufacturer's protocol. Briefly, K562 cells (10^5^ cells/well) were plated in six-well plates the day before transduction. Cells were transduced with either five different shRNA clones targeting KEAP1 or a scrambled non-target control (Scr, SHC002V) in several multiplicity of infections (MOI) to get maximum transduction efficiency in the presence of hexadimethrine bromide (8 *μ*g/mL) and incubated overnight. Cells containing viral-particles were replaced with fresh complete media the next day and allowed to grow overnight. To select the shRNAi transduced cells, media were removed and replaced with complete media containing 2 *μ*g/mL puromycin (Invitrogen). Puromycin-resistant cells containing transduced clones were grown for several passages and tested for *KEAP1* expression by qPCR to determine maximum silencing efficiency (data not shown). The most effective clone (C1) was then selected and maintained in complete media supplemented with puromycin to perform downstream qPCR experiments.

### 2.6. Western Blot

K562 cells were grown in 150 mm dishes and treated with 0.1% DMSO or 10 *μ*M SFN for 5 hours. In addition, K562 shRNA KEAP1-silenced cells from two different clones (C1 and C2) as well as the non-target shRNA control (Control) were grown and collected for protein. Total protein was isolated by incubating cell pellets in RIPA buffer (Thermo Scientific) with 1X protease inhibitors (Roche) for 2 hours on ice, spun at 13,000 rpm (4°C, 30 min), and the protein extracts were quantified by Bradford assay (Bio-Rad). We electrophoresed 30 *μ*g of total protein along with several protein marker ladders (Thermo Scientific) on a 4%–20% gradient Mini-PROTEAN TGX Precast Gel (Bio-Rad) and then transferred onto a 0.45 *μ*M nitrocellulose Hybond-C membrane (GE Healthcare). The membrane was blocked with 5% milk and 1X TBST (Sigma; 2 hours, 4°C) and then probed with either 1 : 200 anti-NQO1 mouse monoclonal (Santa Cruz Biotechnologies, sc-16464), 1 : 200 anti-ABCB6 mouse monoclonal (Santa Cruz, sc-365930), 1 : 200 anti-Ferrochelatase mouse monoclonal (Santa Cruz, sc-377277), 1 : 200 anti-SLC48A1 rabbit polyclonal (Santa Cruz, sc-101957), or 1 : 5000 anti-*β*actin mouse monoclonal (Sigma-Aldrich, A5316), in 5% milk in TBST (overnight, 4°C). After washing, secondary antibody incubations occurred with HRP-conjugated anti-rabbit, anti-mouse, or anti-goat (1 : 2500; Bio-Rad, 170–5046 and 170–5047; Santa Cruz sc-2020, respectively) in TBST with 5% milk (1 hour, room temp). After the final washes, membrane was incubated for one minute in ECL solution (GE Healthcare) and exposed to film. All membranes were incubated with *β*-actin as a loading control.

## 3. Results

### 3.1. SFN Induces *NRF2* but not *NFE2* in Erythroid Cells

**Figure 1:**
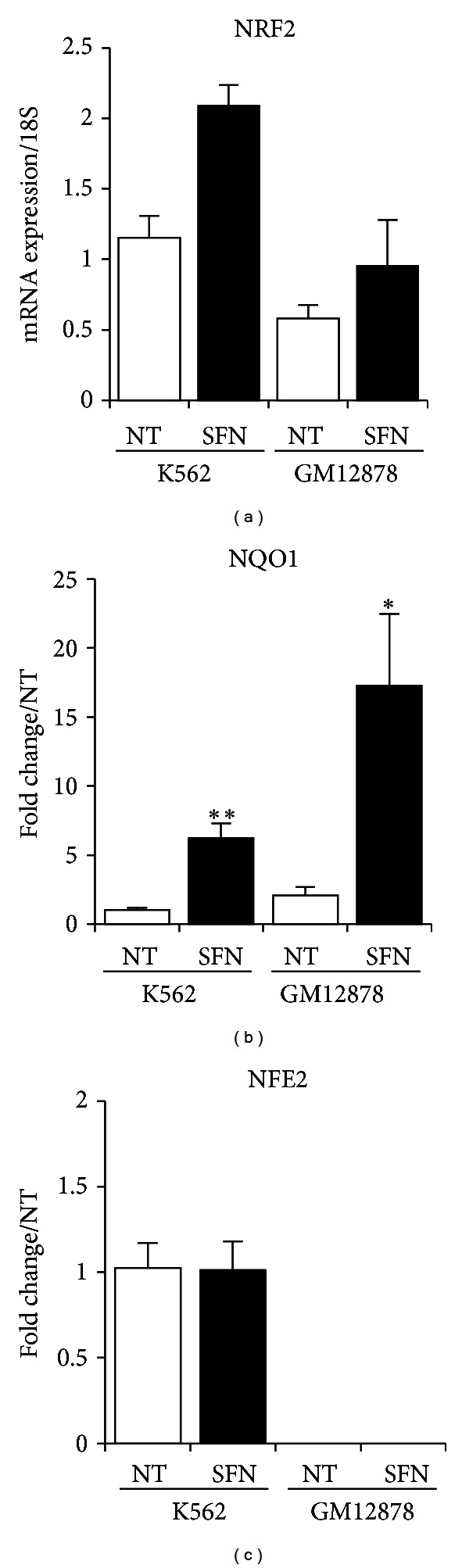
*NRF2*, *NQO1,* and *NFE2 *gene expression in K562 and lymphoid GM121878 cells. (a) *NRF2* mRNA from K562 and GM12878 cells treated for 24 hours with 10 *μ*M SFN increased compared to control. (b) Known NRF2-regulated gene *NQO1* expression significantly increased in both K562 and GM12878 SFN-treated cells. (c) *NFE2-*specific gene expression indicated that *NFE2* was specific to erythroleukemic K562 cells but not inducible upon SFN treatment. Values were normalized to *18S,* and fold change (FC) over nontreated (NT) cells was calculated for (b) and (c). Bars display the average of 3 independent experiments ± SEM; *P < 0.05, **P < 0.01.

In order to establish whether *NRF2* mRNA was inducible by SFN in erythroid cells, K562 and lymphoid GM12878 cells were treated with 10 *μ*M SFN for 24 hours, total RNA was extracted, and qPCR was carried out to measure *NRF2*, the NRF2 target gene *NQO1*, and *NFE2*.  Figure 1(a) shows that *NRF2 *expression was induced by treatment in both lines, and *NQO1* displayed significant 6-fold (K562) and 8.3-fold (GM12878) increases over the respective untreated control (Figure 1(b)). Interestingly, we saw no SFN-induced *NFE2* expression changes in K562 cells and, as expected, little to no *NFE2* expression in lymphoid cells (Figure 1(c)). *NFE2* is known to be exclusively expressed in hematopoietic progenitors and lineages [18]. Although SFN did not induce *NFE2* expression in undifferentiated K562 cells, studies have used dimethyl sulfoxide (DMSO) in murine erythroleukemia (MEL) cells to significantly increase NF-E2 activity which resulted in erythroid differentiation [29]. Testing SFN or other *NRF2* inducers in differentiated erythroid cells would provide additional insight into the inducibility of *NFE2*. 

### 3.2. Lymphoid NRF2 ChIP-seq Peak Regions Colocate with ENCODE K562 NFE2 ChIP-seq Peaks

**Figure 2:**
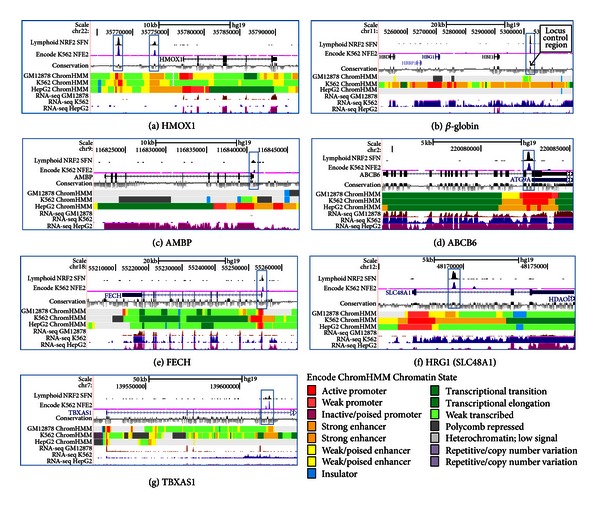
SFN-induced lymphoblastoid NRF2 ChIP-seq, ENCODE chromatin state annotation (ChromHMM), conservation, and ENCODE K562 NFE2 ChIP-seq tracks of NRF2-regulated known and novel target genes. ChromHMM legend shows 15 chromatin state annotations representing the relationship between chromatin state, function, and gene expression. Blue boxes display NRF2, NFE2, and conservation peaks. (a) Known NRF2-regulated *HMOX1*. (b–g) Novel NRF2-bound peak regions colocated with highly conserved ARE sequences and NFE2 ChIP-seq peaks in the (b) *β*-globin locus, (c) *AMBP*, (d) *ABCB6*, (e) *FECH*, (f) *HRG1 (SLC48A1),* and (g) *TBXAS1* genes.

We examined SFN-induced NRF2 binding locations in the *HMOX1* gene using our lymphoid NRF2 ChIP-seq and ENCODE data tracks displayed in UCSC genome browser (Figure 2(a)). In addition to SFN-induced lymphoid NRF2 and untreated K562 NFE2 ChIP-seq tracks, we display ENCODE/Broad tracks showing chromatin state segmentation using the Hidden Markov Model (ChromHMM), RNA-seq profiles, as well as an evolutionary conservation track using the Genomic Evolutionary Rate Profile (GERP) [30] (Figure 2). ChromHMM profiles for GM12878 (lymphoid), K562 (erythroid), and HepG2 (hepatic) are based on the 15 chromatin state annotations determined by multiple histone ChIP-seq experiments and indicate the relationship between chromatin state, DNA function (e.g., active promoter, enhancer, repressed), and gene expression [27]. All three cells lines displayed open active promoters (red), strong enhancers (orange) under the 5′ distal binding peak, a strong enhancer at the more proximal peak, and active transcription (green/lt green) in the gene body. The *HMOX1* genomic region revealed two occupied sites with colocated ChIP-seq peaks (blue boxes) for lymphoid NRF2 and erythroid NFE2, strongly suggesting that these related transcription factors share identical binding sites and binding motifs (Figure 2(a)). In addition, the conservation track showed strong conservation and the response element displays perfect human/rodent homology. RNA-seq tracks indicated slightly more expression in HepG2 cells as reflected by the transcription association state (green) compared to the weak transcription (light green) seen in both the ChromHHM GM12878 and K562 track. Display of the lymphoid NRF2 occupancy and ENCODE tracks allowed a comprehensive visual assessment of known and novel NRF2-occupied regions.

### 3.3. NRF2 Binds the Globin Locus

It has been well established that NFE2 is essential in regulating the beta-globin (*β*-globin) locus control region resulting in activation of **β*-globin* gene expression and erythroid differentiation [18, 31, 32]. In erythroleukemic NFE2-null CB3 cells, erythroid differentiation results in low adult globin gene expression [33]. Additionally, a majority of p45 NFE2 knockout mice die from hemorrhage with surviving adults displaying a mild anemia, including lower hemoglobin levels [18, 34] and sensitivity to oxidative stress [35]. Interestingly, *Nrf2*-deficient mice from various backgrounds also display a hemolytic anemia phenotype [9, 36]. We observed binding of NRF2 in the highly conserved globin locus control region in lymphoblast cells, a novel finding (Figure 2(b)). SFN-induced NRF2 occupancy in lymphoid cells and NFE2 in K562 cells were colocated (blue box) in a region adjacent to fetal (*HBG1*) and embryonic (*HBE1*) globin. Among lymphoid, erythroid, and hepatic cells, vast differences were seen in chromatin states and RNA-seq expression. Specifically, GM12878 and HepG2 cells were completely devoid of expression and displayed repressive heterochromatin (grey) and insulator (CTCF, blue) regions while the K562 ChromHMM track indicated enhancers (orange) with some active promoter (red) and poised (yellow) enhancer regions. High occupancy of this globin control region enhancer by NRF2 in lymphoid cells that did not express *NFE2* suggested a possible role for NRF2 in erythroid cells. NRF2 activation and inducible binding has been suggested as a mechanism to increase expression of fetal **γ*-globin* genes [37], and therefore *NRF2* is being assessed as a potential therapeutic target for modifying sickle cell disease, in which the adult *β*-globin protein is mutant and undergoes hemolysis [37, 38].

### 3.4. Validation Methods for Candidate Genes

**Figure 3:**
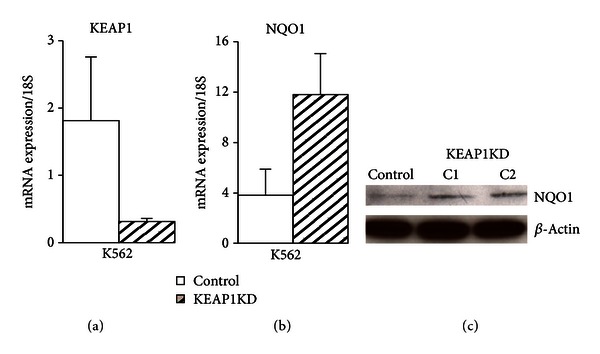
Stable silencing of KEAP1 (KEAP1KD) in K562 cells. (a) KEAP1KD cells showed an 82.7% decrease in *KEAP1* gene expression compared to scrambled control. (b) Basal levels of NRF2-mediated target *NQO1* increased 3.2-fold in KEAP1KD cells. Values were normalized to *18S* and bars represent an average of 3 independent experiments ± SEM. (c) Total protein from KEAP1KD C1 and C2 cells showed an increase in NQO1 compared to control. A NQO1 band at 31 kDA was confirmed by protein ladders, and the membrane was reprobed with *β*-actin as a loading control.

Based on our previous lymphoid NRF2 ChIP-seq data [17], five potential NRF2-regulated genes involved in heme metabolism and iron homeostasis were selected and evaluated in erythroid K562 cells. In order to determine the impact of NRF2 binding in these novel hematopoietic genes, NRF2's cytosolic repressor KEAP1 was stably silenced in K562 cells using shRNA (see Section 2). Inhibiting expression of NRF2's repressor increases the amount of NRF2 available to translocate into the nucleus and increase induction of downstream targets.  Figure 3(a) displayed *KEAP1* gene expression levels in vehicle control and in *KEAP1* knockdown (KEAP1KD) K562 cells. We effectively silenced *KEAP1* expression 82.7% compared to control K562 cells in three independent experiments. In order to determine the impact of silencing KEAP1 in erythroid cells, we ran qPCR, as well as western blot, for the NRF2-mediated gene *NQO1*.  Figure 3(b) indicated that *NQO1* expression in K562 KEAP1KD cells increased 3.2-fold over control. In addition, Figure 3(c) western analysis of K562 KEAP1 silencing revealed an increase in NQO1 protein from two different KEAP1-silenced clone cells lines indicating that NRF2 downstream gene targets were induced in KEAP1-silenced cells allowing us to examine NRF2*-*mediated regulation of hematopoietic gene targets in erythroid cells.

### 3.5. AMBP

**Figure 4:**
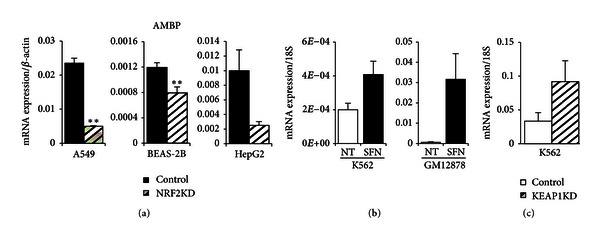
*AMBP* mRNA gene expression in NRF2KD, K562, GM12878, and K562 KEAP1KD cell lines. (a) NRF2 silencing in A549, BEAS-2B, and HepG2 cells treated for 8 hours with 10 *μ*M SFN showed significant decrease in *AMBP* expression suggesting regulation by NRF2. (b) *AMBP* expression levels increased in K562 and lymphoid GM12878 cells treated with 10 *μ*M SFN for 24 hours. (c) Basal levels of *AMBP* increased in K562 KEAP1KD cells indicating NRF2 regulation of *AMBP*. Gene expression values were normalized to *BACT* or* 18S*. Bars represent an average of 3–6 independent experiments ± SEM; *P < 0.05, **P < 0.01.

*AMBP* is a gene that encodes two different proteins, *α*_1_-microglobulin (A1M) and bikunin upon peptide cleavage in hepatocytes, which are then secreted into the plasma [39, 40]. A1M is a 26 kDA glycoprotein belonging to the Lipocalin family with immunoregulatory characteristics and is involved in heme catabolism [41]. Bikunin is a Kunitz-type serine protease inhibitor of the inter-*α*-inhibitor family and a structural component of the extracellular matrix [23, 42]. A1M acts as a reductase and radical scavenger, binding heme in plasma, extravascular fluids, and cells protecting the cell from oxidative damage caused by hemoglobin and heme. [23, 43, 44]. In K562 cells, it has been shown to prevent intracellular oxidation and the upregulation of HMOX1 induced by heme, hydrogen peroxide, and hydroxyl radicals [23]. Figure 2(c) displays colocating peaks in SFN-treated lymphoid and K562 cells for NRF2 and NFE2, respectively (blue box). It was interesting that NRF2 and NFE2 displayed occupancy in the *AMBP* promoter despite the presence of repressive chromatin (grey) and insulator (blue) regions in ChromHMM profiles for lymphoid and erythroid cells. In contrast, HepG2 chromatin indicated open active promoters (red), enhancers (orange), and active transcription (green) which was supported by the RNA-seq data for this region of the *AMBP* gene. In order to confirm that NRF2 was regulating *AMBP* gene transcription, we transiently silenced NRF2 in airway (BEAS-2B and A549) and liver (HepG2) cell lines. These lines are common *in vitro* models used to examine cellular response to oxidative stress exposure. The level of SFN-treated, NRF2-silenced (NRF2KD) *AMBP* mRNA was significantly reduced when compared to the SFN-treated control by 78.6% (BEAS-2B), 33.9% (A549), and 74.9% (HepG2), indicating that *AMBP* transcription was regulated by NRF2 (Figure 4(a)). Next we compared *AMBP* gene expression in untreated and SFN-treated K562 and lymphoblastoid GM12878 cell lines. K562 cells showed a modest 2-fold *AMBP* induction compared to the 56-fold increase over no treatment in GM12878 cells, indicating that SFN was a stronger inducer of *AMBP* in lymphoid cells (Figure 4(b)). Additionally, we created stable silencing of NRF2's cytosolic repressor KEAP1 in erythroid K562 cells and assessed them for *AMBP* inducibility. A 2.75-fold increase over control indicated NRF2 may be binding and affecting the regulation of *AMBP* (Figure 4(c)). Other findings [23] suggest that *AMBP* may be an antioxidant that binds and degrades heme in erythroid lines, and the present study suggests that this function may be under the regulation of NRF2.

### 3.6. ABCB6

**Figure 5:**
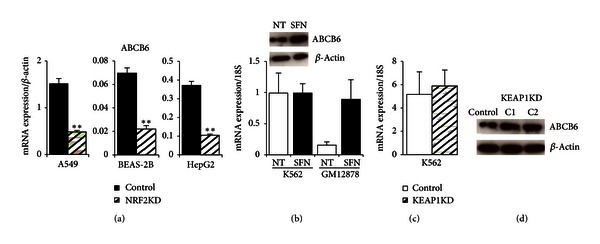
Gene expression and protein analysis of *ABCB6* in NRF2KD, K562, GM12878, and K562 KEAP1KD cells. (a) Significant *ABCB6* decrease in NRF2-silenced A549, BEAS-2B, and HepG2 cells treated for 8 hours with 10 *μ*M SFN. (b) *ABCB6 *was not induced by 24 hours of 10 *μ*M SFN treatment in K562 cells but responded to chemical treatment in lymphoid GM12878 cells. Protein levels of ABCB6 show an increase after treatment of K562 cells with 10 *μ*M SFN for 5 hours compared to respective controls. (c) Similar to (b), high baseline levels of *ABCB6* mRNA did not change significantly in K562 KEAP1KD cells. Gene expression values were normalized to *BACT* or* 18S*. Bars represent an average of 3–6 independent experiments ± SEM; *P < 0.05, **P < 0.01. (d) KEAP1KD C1 and C2 total protein show increased levels of ABCB6. Protein markers confirmed an ABCB6 band at 79 kDA, and the membrane was reprobed with *β*-actin as a loading control.

Lymphoid NRF2 binding was seen in *ABCB6*, a putative NRF2 target gene first identified in microarray analysis of human small airway epithelium cells from healthy smokers [45]. ABCB6 is a porphyrin transporter located at the outer mitochondrial membrane in RBCs and has been suggested to translocate CPgenIII from the cytoplasm to the mitochondria, an important step in the sixth enzymatic reaction in the heme biosynthesis pathway [46]. It is highly expressed during erythroid differentiation and controls the translocation of heme and heme precursors between the cytoplasm and mitochondria [25, 44]. The *ABCB6* gene has also been shown to encode the Lan blood group antigen which causes severe hemolytic transfusion reactions [47]. In K562 cells, exogenous expression of *ABCB6* resulted in cell surface detection of Lan antigen [46, 48]. It has recently been located not only on the plasma membrane on RBCs but also at the plasma membrane of HepG2 hepatocellular carcinoma (HCC) cells which may play a role in drug resistance in HCC and other cancer cells [47]. NRF2 occupancy was colocated with NFE2 peaks (Figure 2(d), blue box) in the *ABCB6* gene with almost perfect human to mouse homology of the ARE sequence. ChromHHM tracks under the peaks displayed active open promoters (red) and active transcription (green), as supported by RNA-seq data, in lymphoid GM12878, erythroid K562, and hepatic HepG2 cell lines. In NRF2-silenced BEAS-2B, A549, and HepG2 cells, SFN-induced *ABCB6* expression was significantly reduced by 68.2%, 68.1%, and 71.7% of control mRNA (Figure 5(a)). Comparing *ABCB6* expression in K562 and GM12878 cells treated with SFN for 24 hours showed no SFN-induced changes in K562 cells and a 5.8-fold increase over control in GM12878 cells (Figure 5(b)). Interestingly, five-hour SFN treatment of K562 cells increased ABCB6 protein compared to untreated cells. Silencing KEAP1 in K562 cells did not have a significant effect on *ABCB6* mRNA expression, although K562 cells had a very high baseline expression level in untreated and non-target shRNA conditions (Figures 5(b) and 5(c)).

Examining ABCB6 protein levels in K562 KEAP1KD from two different silenced cell lines and their respective controls, we observed that both C1 and C2 clones showed an increase in ABCB6 compared to the control (Figure 5(d)). Thus, ABCB6 responded to SFN treatment, and ABCB6 protein appears higher in the KEAP1KD cells. In summary, NRF2 may be regulating *ABCB6* in lymphoid cells as well as in erythroid cells. Using a different model system, such as primary differentiating hematopoietic cells, would present an additional method to examine the impact of NRF2 binding in the *ABCB6* gene.

### 3.7. FECH

**Figure 6:**
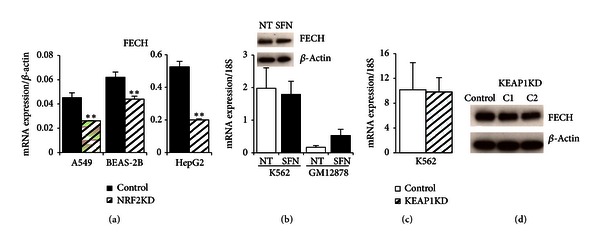
NRF2-silenced cell lines, erythroid, lymphoid, and KEAP1-silenced protein and mRNA expression of *FECH*. (a) *FECH* was significantly decreased in SFN-induced NRF2-silenced mRNA from airway and hepatic cell lines. (b) K562 cells did not respond to chemical treatment at mRNA (10 *μ*M SFN, 24 hours) or protein level (5 hours with 10 *μ*M SFN), unlike the SFN-mediated increase of *FECH* in lymphoid GM12878 cells. (c) K562 KEAP1KD FECH mRNA was unchanged compared to control. Gene expression values were normalized to *BACT* or* 18S*. Bars represent an average of 3–6 independent experiments ± SEM; *P < 0.05, **P < 0.01. (d) FECH protein did not increase in K562 KEAP1-silenced cells. Protein markers confirmed FECH monomers at ~40 kDA, and the membrane was reprobed with *β*-actin as a loading control.

Ferrochelatase (FECH) is an enzyme localized in the mitochondria where it is responsible for the last step of heme biosynthesis, catalyzing the insertion of the ferrous form of iron to protoporphyrin IX [25, 49]. It was proposed that FECH interacts with mitoferrin-1 (MFRN1), a mitochondrial iron uptake protein that is upregulated during erythroid differentiation [50]. In addition, recent studies suggested that FECH forms a macromolecular complex with MFRN1 and ABCB10 during erythroid differentiation [50–52]. *FECH* gene expression level has been associated with erythropoietic protoporphyria (EPP) in humans [53]. In human trials following dietary ingestion of SFN, NRF2-mediated genes such as *NQO1* and *HMOX1* displayed induced gene expression (200%–300% increase in epithelial tissues) [54]. Therefore, it was of interest to further examine *FECH* as an NRF2 regulated gene. NRF2 and NFE2 ChIP-seq peaks colocated (blue box) in the conserved ARE consensus sequence of the *FECH* gene (Figure 2(e)). Chromatin status indicated open promoters (red) with relatively weak transcription (lt. green) in GM12878 and HepG2 compared to stronger transcription levels (green) of *FECH* in the K562 cells. *FECH* mRNA levels were relatively low in A549 and BEAS-2B cells (Figure 6(a)). However, *FECH* mRNA expression was ~100-fold higher in HepG2 cells compared with A549 or BEAS-2B cells, and it was induced 3-fold by SFN in LCLs (Figure 6(b)). NRF2 silencing reduced SFN-induced levels of *FECH* expression significantly by 42.2% (A549), 29.3% (BEAS-2B) and 61.9% (HepG2) compared to cell line specific controls (Figure 6(a)). However, *FECH* expression in K562 cells did not change with SFN treatment (either at mRNA or protein level) but increased 3.1-fold in lymphoid cells (Figure 6(b)). The very high baseline level of *FECH* expression in K562 may preclude inducibility, even if NRF2 is upregulated as in K562 KEAP1KD cells (Figure 6(c)). FECH protein levels did not change in the K562 KEAP1KD clones (Figure 6(d)). Previously, studies have shown that *FECH* is highly upregulated during erythroid differentiation [50] and in hemin-stimulated differentiating K562 cells [55]. A model system that examined NRF2 mediation of *FECH* expression under differentiation conditions may allow further insight into this relationship.

### 3.8. HRG1 (SLC48A1)

**Figure 7:**
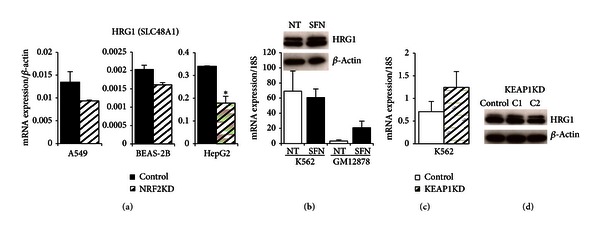
*HRG1 (SLC48A1) *gene expression and protein analysis in NRF2KD, K562, GM12878, and K562 KEAP1KD cells. (a) Transient silencing of NRF2 in three cell lines (A549, BEAS-2B, and HepG2) reduced expression of *HRG1*. Most significant *HRG1 *reduction was observed in HepG2 followed by A549. (b) Chemical activation of NRF2 using SFN (10 *μ*M, 24 hours) induced *HRG1* expression in GM12878, but high *HRG1* baseline levels remained unchanged in SFN-induced K562 cells. K562 cells treated with 5 hours of 10 *μ*M SFN showed an increase in HRG1 protein indicating a response to chemical activation at the shorter timepoint. (c) Genetic activation of NRF2 by stably silencing its negative regulator KEAP1 induced *HRG1* in K562 cells at mRNA and protein level. (d) Two different shRNA clones showed an increase in HRG1 as compared to non-target shRNA control. mRNA values were normalized to *BACT* or* 18S*. Bars represent an average of 3–6 independent experiments ± SEM; *P < 0.05, **P < 0.01. A 30 kDA HRG1 band was confirmed by protein markers, and the membrane was stripped and reprobed with *β*-actin as a loading control.

Initially discovered in *C. elegans*, heme responsive gene 1 (*HRG1*) was the first heme importer identified and is the only member of the solute carrier 48 (SLC48) family [48, 49, 56]. Located in the plasma and lysosomal membrane, HRG1 transports heme from the lysosome into the cytoplasm and is essential for normal development [25]. In addition, knockdown of *HRG1* in zebrafish resulted in severe anemia and hydrocephaly [48]. As with the other genes we examined, NRF2 and NFE2 co-occupied the same *HRG1* genomic region (blue box) in lymphoblastoid and K562 cells suggesting that they may have an overlapping regulatory role in the expression of *HRG1* (Figure 2(f)). Comparisons of ChromHMM and RNA-seq tracks for the three cell lines indicate that *HRG1* was highly expressed in K562 cells and shows an active promoter (red), strong enhancer (orange), and transcriptional elongation (green) chromatin states (Figure 2(f)). Unlike K562 cells, expression of *HRG1* in GM12878 was very low, and this was consistent with observed ChromHMM states of weak promoters (light red), weak poised enhancers (yellow), and a large insulator region (blue; Figure 2(f)). The expression of *HRG1* was reduced in all three cell lines in which *NRF2* was silenced transiently but was most strongly reduced in HepG2 (52.5%) suggesting cell-type specific regulation of *HRG1* by NRF2 (Figure 7(a)). Upregulation of mRNA expression of *HRG1* was observed both in response to SFN treatment (6.5-fold induction) and genetic activation in K562 (1.8-fold induction) (Figures 7(b) and 7(c)). Protein levels for HRG1 showed small increases for both chemical and genetic NRF2 activations (Figures 7(b) and 7(d)).

### 3.9. TBXAS1

**Figure 8:**
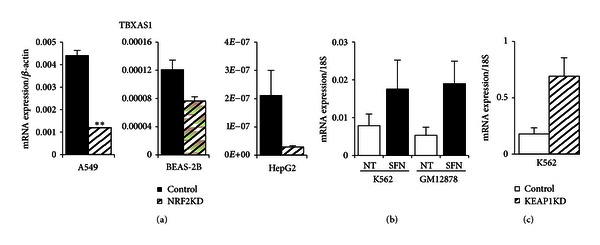
*TBXAS1* gene expression in airway, hepatic, erythroid, and lymphoid cell lines. (a) NRF2 silencing in three cell lines (A549, BEAS-2B, and HepG2) treated for 8 hours with 10 *μ*M SFN. *TBXAS1* expression was reduced most significantly in A549 followed by HepG2 cells. (b) Chemical activation (10 *μ*M SFN for 24 hours) of NRF2 in K562 and GM12878 cells led to robust induction of *TBXAS1*. (c) Genetic activation of NRF2 by KEAP1 silencing led to a potent *TBXAS1* activation in erythroid K562 cells. Values were normalized to *BACT* or* 18S*. Bars represent an average of 3–6 independent experiments ± SEM; *P < 0.05, **P < 0.01.

*TBXAS1 *(cytochrome P450, family 5, subfamily A, *Cyp5A1*) is considered a member of the cytochrome P450 enzyme family based on sequence homology but is functionally very different from this group of monooxygenases and quite different from the other candidate genes. TBXAS1 resides in the endoplasmic reticulum membrane and functions as a catalyzer for the conversion of prostaglandin H2 to thromboxane A2, which is a potent vasoconstrictor, bronchoconstrictor, and inducer of platelet aggregation [57, 58]. In addition to its role in several pathophysiological processes including hemostasis, recent research suggested a role for *TBXAS1* in several cancers as well as preeclampsia [59–61]. Because of its biological significance and its occupancy by NRF2 in lymphoblastoid cell lines, we further explored NRF2-dependent expression. Occupied binding sites were seen in NRF2 and NFE2 ChIP-seq experiments in GM12878 and K562 cells, respectively, indicating potential regulation by these transcription factors (Figure 2(g), blue box). Large differences in ChromHMM tracks were seen among the three cell lines reflecting the RNA-seq expression patterns shown (Figure 2(g)). HepG2 cells showed a repressed state while GM12878 and K562 cells showed strong enhancers (orange) under NRF2 and NFE2 binding sites. K562 cells, however, showed increased transcription (green) compared to lt green (weak transcription) and grey (repression) states in GM12878 cells resulting in higher expression of the *TBXAS1* gene in K562 cells. Unlike the expression pattern of *HRG1*, both chemical and genetic activations of NRF2 led to the upregulation of *TBXAS1* in K562 cells (Figures 8(a)–8(c)). The genetic activation of NRF2 in K562 cells by silencing KEAP1 resulted in a 3.9-fold induction of *TBXAS1* compared to 2.2-fold induction with SFN treatment (Figures 8(b) and 8(c)). Transient silencing of *NRF2* in all three cell lines reduced the expression of *TBXAS1* with varying degree (35%–87%) suggesting that *TBXAS1* was a potential direct target of NRF2 (Figure 8(a)). In aggregate, for each of these candidate genes we see evidence of NRF2 occupancy in enhancer regions that is colocated with NFE2 occupancy in K562 cells, as well as NRF2-dependent gene expression in one or more of the experimental systems tested. 

## 4. Discussion

It is well known that NRF2 mediates the regulation of several genes involved in iron metabolism (*FTL, FTH1*) and the heme metabolic pathway (*HMOX1*) [10, 17]. NRF2 also plays a critical role in the survival of RBCs by regulating intracellular ROS levels when selenoproteins, enzymes with antioxidant properties, are depleted [9]. Here, we selected five novel NRF2- regulated hematopoietic genes based on NRF2 ChIP-seq in lymphoid cells and investigated their NRF2-dependent regulation using silencing techniques and activation treatments. In order to address NRF2-mediated expression of these hematopoietic genes in an erythroid line, K562 cells were treated with SFN to activate NRF2 and compared with lymphoid cells. Gene expression of candidate NRF2-mediated targets increased upon SFN treatment in lymphoid cells consistent with SFN-induced NRF2 occupancy detected by ChIP-seq. In addition, *AMBP* and *TBXAS1* expression were increased both upon chemical induction by SFN and in KEAP1-silenced K562 cells. *ABCB6*, *FECH,* and *HRG1* expression showed high baseline values in K562 and were not inducible by SFN at the 24 hr SFN timepoint. However, both ABCB6 and HRG1 protein levels increased in a five-hour SFN exposure as well as in the KEAP1-silenced condition. *ABCB6* expression was not induced in SFN-treated A549 or BEAS2-B cells but did increase in HepG2 cells (data not shown). In addition, NRF2 silencing in untreated cells showed a reduction (50%–73%) in all three cells lines [17] suggesting NRF2 regulation at both a basal and inducible level for *ABCB6*. *FECH* expression showed a similar pattern with 24-hour SFN treatment, but A549 and BEAS-2B did not change with NRF2 silencing in untreated cells [17], unlike all three silenced SFN-treated cell lines (Figure 6(a)) suggesting the possibility of cell type-specific regulation of FECH by NRF2. *HRG1* expression patterns were unchanged in all three untreated NRF2-silenced cell lines compared to control, yet SFN treatment varied among the cell lines (data not shown) suggesting the possibility of inducible regulation by NRF2. Using primary erythroid differentiating cells may be a better model to investigate NRF2 regulation of *ABCB6*, *FECH,* and *HRG1 *since all three genes are developmentally essential and are highly upregulated during erythroid differentiation [25, 49, 62]. 

One novel finding was lymphoid SFN-induced NRF2 peaks colocating at the same ARE consensus sequence as erythroid NFE2 in all of the hematopoietic candidates as well as the *β*-globin control region locus. Lymphoid cells do not express NFE2 suggesting a possible interaction between NRF2 and NFE2 or a response to oxidative stress in erythroid cells at NFE2 enhancer sites. NRF2 and NFE2 bind the same consensus sequence and heterodimerize the same small Maf proteins (MafF, MafG, MafK), possibly allowing either transcription factor to regulate the hematopoietic genes depending on cellular context, differentiation state, or redox homeostasis. In differentiating MEL cells, for example, MafK, a corepressor and binding partner of Bach1, shifted dimerization partners to become a coactivator with Nfe2 and bind at the *β*-globin locus control region [63]. Studies suggest that BACH1 heterodimerizes the same small Maf and other bZIP proteins as NRF2 and NFE2, indicating an interactive competition for small Maf proteins [64]. A study by Sun et al. [65] using multiple ChIP assays in preadipocyte mouse 3T3 cells demonstrated that increasing heme levels caused displacement of Bach1 from MafK heterodimers at the Hmox1 enhancer, allowing Nrf2 to heterodimerize with MafK in the same response element. Therefore, NRF2 may be responding to cellular stress in erythroid cells by heterodimerizing with Maf proteins and binding NFE2 enhancer sites in the promoters of genes that can modify the response to oxidative damage. 

NRF2 mediation of heme metabolism and iron homeostatis genes in response to oxidative stress would be beneficial to cell survival. In K562 cells, for example, AMBP is induced by heme, hemoglobin, and ROS, and this change is accompanied by upregulation of HMOX1 leading to prevention of intracellular oxidative damage [23]. Under conditions of phenylhydrazine-induced stress, *ABCB6* was found to be essential to survival in ABCB6 knock-out mice as the sole ATP-dependent porphyrin importer [62], and it seems likely that NRF2-dependent upregulation of porphyrin transport under oxidative stress conditions would be protective as well. Similarly, upregulation of *FECH* under oxidative stress conditions would increase production of functional heme and to support higher levels of oxygen transport. HRG1 was proposed to be responsible for transporting endocytosed heme out of the phagosome and into the cytosol for subsequential degradation by HMOX1 [66], and coordinated regulation of these two genes seems likely. While TBXAS1 is functionally quite different from the other candidates, changes in TBXAS1 expression in lung cancer cell lines have been associated with increased ROS production and induction of apoptosis, a finding that was negated when treated with antioxidant treatment [60]. Taken together, NRF2 mediation of heme metabolic and hemostatic genes *AMBP, ABCB6, FECH, HRG1,* and *TBXAS1* provides additional insight into the vast functional diversity of NRF2 transcriptional control.
